# Incidence, Time Course and Predictors of Impairments Relating to Caring for the Profoundly Affected arm After Stroke: A Systematic Review

**DOI:** 10.1002/pri.1634

**Published:** 2015-05-25

**Authors:** Rhoda Allison, Laura Shenton, Kathryn Bamforth, Cherry Kilbride, David Richards

**Affiliations:** ^1^ Stroke Service, Newton Abbot HospitalTorbay and Southern Devon Health and Care TrustNewton AbbotUK; ^2^ Mood Disorders Centre, College of Life and Environmental SciencesUniversity of ExeterExeterUK; ^3^ Brunel University LondonCentre for Research in RehabilitationLondonUK

**Keywords:** disability, pain, stroke, spasticity

## Abstract

**Background and purpose:**

A significant number of stroke survivors will not recover the use of their affected arm. A proportion will experience pain, stiffness and difficulty with basic care activities. The purpose of the review was to identify predictors of difficulty caring for the profoundly affected arm and establish the incidence and time‐course of the related impairments of pain, spasticity and contracture.

**Method:**

Data sources: Databases (PubMED, MEDLINE, AMED, EMBASE, CINAHL and the Cochrane Controlled Trials Register) were searched from inception to December 2013. Additional studies were identified from citation tracking. Review methods: Independent reviewers used pre‐defined criteria to identify eligible studies. Quality assessment and risk of bias were assessed using the McMasters Assessment Tool. A narrative evidence synthesis was performed.

**Results:**

Thirty‐nine articles reporting 34 studies were included. No studies formally measured difficulty caring for the arm, but related impairments were common. Incidence of spasticity in those with weakness ranged from 33% to 78%, shoulder pain affected 22% to 90% and contracture was present in at least 50%. Spasticity and pain appear within 1 week of stroke, and contracture within two weeks. Impairments continued to develop over at least 3–6 months. The most frequent predictors of spasticity and contracture were weakness and reduced motor control, and the risk of pain is most commonly predicted by reduced sensation, shoulder subluxation, weakness and stroke severity.

**Discussion:**

There is no published evidence on predicting the likelihood of difficulty caring for the arm following stroke. However, the related impairments of spasticity, pain and contracture are common. Given the time‐course of development, clinicians may need not only to intervene early but also be prepared to act over a longer time period. Further research is needed to examine difficulty caring for the arm and the relationship with associated impairments to enable researchers and clinicians to develop targeted interventions. © 2015 The Authors. *Physiotheraphy Research International* Published by John Wiley & Sons Ltd.

## Introduction

Stroke is the second largest cause of death in adults and the principal cause of long‐term severe adult disability worldwide (Lopez and Mathers, 2006; American Heart Association, 2009; Department of Health, 2009). Seventy per cent of people with stroke will experience arm weakness, and 62% of these will not recovery dexterity in the arm at 6 months post‐stroke (Kwakkel et al., 2003). For the purposes of this review, the term ‘profoundly affected arm’ is used to describe the situation where a stroke survivor has no movement in the affected arm or when movement is not functionally useful. This term was developed in consultation with a group of stroke survivors.

Current physical therapies in stroke rehabilitation are based predominantly on exercise and task‐specific training (Duncan et al., 2005; Intercollegiate Stroke Working Party, 2012). However, most interventions aimed at improving active function require the presence of some movement within the arm initially, and research has shown that additional physiotherapy and practice of motor tasks do not improve active function in those with most significant arm weakness (Parry et al., 1999). For those unlikely to regain active function, a different approach focused on managing disability and avoiding complications in the arm is required. Managing disability involves assessing and reducing impairments in the arm, which can impact negatively on the ability to care for the arm including tasks such as hand washing, nail cutting and dressing (passive function activities) (Sheean, 2001). Impairments, which are commonly associated with the profoundly affected arm and are often targeted for treatment in order to reduce difficulty caring for the arm, include spasticity (Bhakta et al., 2000), contracture (De Jong et al., 2006, 2006) and pain (Shaw et al., 2010). People with arm spasticity and contracture may develop abnormal limb posturing, which can make washing of the axilla, elbow crease and hand difficult, leading to hygiene problems, and potential skin breakdown (Mayer et al., 1997; Fergusson et al., 2007) and increased carer burden (Katalinic et al., 2010). Equally, pain is also often a focus of treatment in improving care of the arm (Ashford and Turner‐ Stokes, 2009). It is possible that other impairments may impact on passive function of the arm, but there is currently little evidence to support a positive relationship between complications in the arm and impairments such as joint subluxation (Kumar and Swinkels, 2009), and it is difficult to assess the impact of sensory changes in isolation from motor problems (Intercollegiate Stroke Working Party, 2012).

Management of the profoundly affected arm is a complex intervention, which has traditionally included techniques such as splinting (Lannin et al., 2007), positioning (De Jong et al., 2006), stretching (Bovend'Eerdt et al., 2008) and the use of medications such as botulinum toxin (Bhakta et al., 2000). However, evidence to support these interventions is mixed, and frequently, trials have been designed without considering the natural course of impairments and disability in this condition. For example, in a study of splinting to prevent contracture (Lannin et al., 2007), the intervention was provided within the first 8 weeks of stroke for a period of 4 weeks, but there was no rationale to suggest if these timings reflect the period where risk of contracture is greatest (Manigandan and Charles, 2007). Currently, little is known about which people with a profoundly affected arm are most at risk of developing associated impairments or difficulty with passive function. This systematic review has two aims. Firstly, to identify the incidence and natural course of pain, spasticity, contracture and difficulty with passive care in the profoundly affected arm. Secondly, to identify potential predictors that could be used in routine clinical settings in the early stages of post‐stroke care to identify those most at risk of difficulty caring for the arm or these related impairments. This is important, as more knowledge of how the profoundly affected arm changes over time will assist researchers and clinicians in designing and evaluating appropriately timed and targeted interventions to ultimately benefit the stroke survivor and their carers.

## Methods

### Search strategy

The following databases were systematically searched: PubMED, MEDLINE, EMBASE, CINAHL, AMED and the Cochrane Library, from the inception date of each database up to December 2013. The search terms are given in Appendix 1. In addition, citation tracking of journals was undertaken.

#### Criteria for inclusion of studies

The review included published research articles that fulfilled the following PICOS (Liberati et al., 2009) criteria:

Participants: adults (over 18 years of age) with arm weakness post‐stroke
Interventions: the review was not designed to evaluate a specific intervention but did not exclude reports of data from intervention studies that provided data to answer the review questions (for example, data from control groups identifying changes over time)
Comparators: not applicable
Outcomes: ease or difficulty of passive function of the arm, pain, spasticity or contracture.
Study design: (1) Observational studies of the natural course of events post‐stroke and (2) studies evaluating the ability of identified factors (either demographic factors or impairments related to post‐stroke presentation) that were assessed within the first 8 weeks of stroke to predict pain, impairment and capacity to care for the arm after stroke.

Studies were excluded if they were not available in English, targeted children or if purely laboratory‐based tests such as medical imaging used as predictors. Case series and case reports were excluded owing to the high potential for bias in these study designs. Studies that considered recovery of active function in the arm only were also omitted.

### Study selection

Initially, titles, then abstracts, were screened by two members of the review team, working independently. Full studies that met the inclusion criteria were obtained for more detailed evaluation.

### Data extraction, management and assessment of potential risk of bias

Two reviewers, working independently, undertook the data extraction and identification of risk of bias, using structured formats. Key data extraction included the following items: general study information (title, author and country of study); study design and characteristics (participant characteristics, potential predictors and outcomes); and findings including length of follow‐up. Agreement between reviewers was calculated using kappa scores, and any differences in data extraction were resolved by mutual agreement, and where necessary, referred to a third person. Quality assessment and risk of bias in the selected studies were appraised using a tool adapted from the Quality Assessment Tool for quantitative studies developed by the Effective Public Health Practice Project at McMaster's University in Canada (Effective Public Health Practice Project, 2008).

### Summary measures and synthesis of results

The principle summary measures were incidence of each impairment and risk ratio for predictors of either impairment or difficulty with passive care (when this was reported). Data were narratively synthesized via a series of summary tables and reported incidence, change over time and results of any evaluation of predictors. Meta‐analysis was not indicated because of inherent heterogeneity of the studies.

## Results

### Study selection

**Figure 1:**
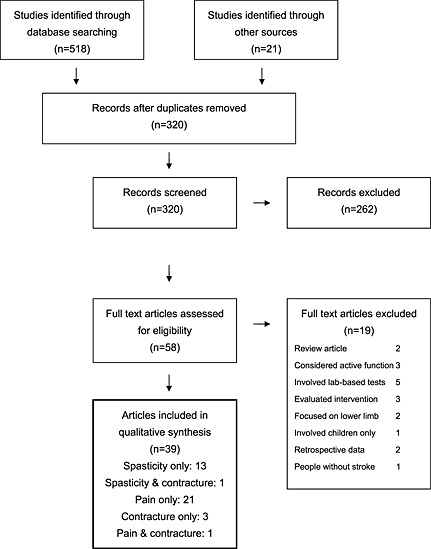
Search results

A total of 539 references were initially identified. There were 219 duplicate references. Figure 1 summarizes the search and reasons for exclusion. Fifty‐eight full articles were retrieved, but a further 19 were excluded because they focused on only active rather than passive function, did not include the arm, evaluated laboratory‐based tests or imaging or included people with arm weakness for other reasons than stroke. In total, 39 publications were suitable for quality assessment. Five pairs of articles (Table 2) presented differing data from the same studies, but to prevent double reporting, this review includes 39 publications, describing 34 different studies.

## Study characteristics

### Participants

The characteristics of study participants are summarized in Table 1. Overall a total of 20,590 patients participated in the studies. None of the studies specifically targeted people with a profoundly affected arm. Broadly, they focused on either general populations of people recovering from stroke (including those with a weak arm) or targeted specific populations including people with stroke and hemiplegia, weakness or those who needed rehabilitation. Five studies limited recruitment to people who had sustained ischaemic stroke only, but the others did not differentiate between people with sub‐types of stroke. One study explicitly included people with more severe stroke moving to care homes (Sackley et al., 2008). Six studies were from the UK, 11 from Europe, 3 from North America and 14 from other countries. One study involved participants in 35 different countries (O'Donnell et al., 2013). The average age of study participants was 65.5 years, and they were recruited at any point between the onset of stroke and 1 year after.

**Table 1:** Characteristics of participants and studies

	Setting	Sample size	Targeted population	Time since stroke at recruitment (days)	Average age at recruitment (years)	Impairment studied	Design
Appelros, 2006	Sweden	253	People with first‐ever stroke	Fixed: onset	74 (33–95)	Pain‐general	Longitudinal
Ada et al., 2006	Australia	18	People with stroke, with hemiplegia	Variable: 17 (14–28)	63 (36–82)	Contracture‐elbow	Longitudinal
Aras et al., 2004	Turkey	85	People with hemiplegia and receiving rehabilitation	Variable: not stated	59.5 (47–70)	Pain‐shoulder	Cross‐sectional
Bohannon, 1988	United States	30	People with stroke, with hemiplegia and receiving rehabilitation	Variable: 31 (SD 15)	68 (SD 10.6)	Pain‐shoulder	Longitudinal
Cheng et al., 1995	Taiwan	50	People with stroke, receiving inpatient rehabilitation	Variable: 21–180	62 (40–79)	Pain‐shoulder	Cross‐sectional
De Jong et al., 2011	The Netherlands	50	People with first ischaemic stroke with arm weakness, receiving TMS	Variable: within 48 hours	70.3 (58–82)	Spasticity‐elbow	Longitudinal
Gamble et al. 2002	UK	123	People with stroke	Fixed: 14	70.6 (29–93)	Pain‐shoulder	Longitudinal
Gamble et al., 2000							
Hadianfard and Hadianfard, 2008	Iran	152	People with stroke	Variable: 0–60	61 (40–75)	Pain‐shoulder	Longitudinal
Kong et al., 2012	Singapore	148	People with stroke, with weakness and receiving rehabilitation	Variable: not reported	63 (53–76)	Spasticity‐arm	Longitudinal
Kong et al., 2010	Singapore	140	People with stroke, with weakness and receiving rehabilitation	Variable: 15 (SD 14.6)	61 (SD 13.3)	Spasticity‐arm	Longitudinal
van Kujik et al., 2007	Holland	40	People with ischaemic stroke, with complete arm paralysis	Fixed: onset	68 (59–77)	Spasticity‐arm	Longitudinal
Kuptniratsaikul *et al.*, 2013	Thailand	214	People with stroke	Variable: (median 24)	62 (59–75)	Spasticity‐general	Longitudinal
Kwah et al., 2012	Australia	165	People with stroke	Variable: up to 28 days	78 (IQR 65–84)	Contracture‐general	Longitudinal
Leathley et al., 2004	UK	106	People with stroke	Fixed: onset	70 (SD 11.3)	Spasticity‐general	Longitudinal
Watkins et al., 2002							
Lindgren et al., 2007	Sweden	327	People with first ever stroke	Fixed: onset	73 (17–102)	Pain‐shoulder	Longitudinal
Lindgren et al., 2012		58 (subset)	People with first ever stroke, with motor or sensory deficit and pain		71		
Lundstrom et al., 2010	Sweden	47	People with first ever stroke and initial weakness	Variable: 2–10 days	74 (34–84)	Spasticity‐arm or leg	Longitudinal
Lundstrom et al., 2009	Sweden	140	People with first stroke	Fixed: 12 months	71 (SD 13)	Pain‐general	Cross
Lundstrom et al., 2008						Spasticity‐arm	sectional
Malhotra et al., 2011	UK	30	People with first stroke and no function of arm	Variable: 21 (range 7–35)	70 (52–90)	Contracture‐wrist	Longitudinal
Moura et al. 2009	Brazil	146	People with ischaemic stroke	Fixed: onset	64 (25–88)	Spasticity‐general	Longitudinal
O'Donnell et al., 2013	35 countries	15754	People with non‐severe ischaemic stroke, over 50 years	Variable: (median 15)	65 (not reported)	Pain‐general	Longitudinal
Paci et al., 2007	Italy	107	People with first stroke and hemiplegia receiving rehabilitation	Variable: 7–27 days	72 (62–82)	Pain‐shoulder	Longitudinal
Pandyan et al., 2003	UK	22	People with stroke, with weakness	Variable: 14–28	65 (40–93)	Contracture‐wrist	Longitudinal
						Spasticity‐ wrist	
Picelli et al., 2013	Italy	72	People with first ischaemic stroke, with hemiplegia and receiving rehabilitation but not receiving medications for spasticity	Variable: within 7 days	71 (SD 10)	Spasticity‐arm	Longitudinal
Pong et al., 2012	Taiwan	76	People with first stroke, with hemiplegia	Variable: not reported	59 (SD 13)	Pain‐shoulder	Longitudinal
Poulin de Courval et al., 1990	Canada	94	People with stroke, with hemiplegia and receiving rehabilitation	Variable: 21–35	Not reported	Pain‐shoulder	Cross‐sectional
Rajaratnam et al., 2007	Singapore	135	People with unilateral stroke	Variable: 2–14	64 (SD 10.8)	Pain‐shoulder	Cross sectional
Ratnasabapathy et al., 2003	New Zealand	1201	People with first ever stroke	Variable: 0–14	Not reported	Pain‐shoulder	Longitudinal
Roosink et al., 2011	The Netherlands	31	People with first ever stroke, with sensory or motor signs	Fixed: 2 weeks	67 (52–82)	Pain‐shoulder	Longitudinal
Sackley et al. 2008	UK	73	People with Barthel score of <10 at 3 months post‐stroke	Fixed: 3 months	76 (31–98)	Pain‐shoulder	Longitudinal
						Contracture‐general	
Sommerfeld et al., 2004 Sommerfeld and Welmer, 2012	Sweden	95	People with first ever stroke	Fixed: onset	78 (SD 9.5)	Pain‐general	Longitudinal
		66 (subset)				Spasticity‐general	
Suethanapornkul and Kuptniratsaikul, 2008	Thailand	327	People with stroke who could sit out of bed for 30 minutes	Variable: 84	62 (50–74)	Pain‐shoulder	Longitudinal
Urban et al., 2010	Germany	211	People with first stroke, with weakness	Variable: not reported	68 (59–78)	Spasticity‐general	Longitudinal
						Spasticity‐arm	
Wanklyn et al., 1996	UK	108	People with stroke, with ongoing disability returning home	Variable: not reported	Not reported	Pain‐ shoulder	Longitudinal
Zorowitz et al. 1996	United States	20	People with stroke presenting with shoulder subluxation	Variable: 13–40	63 (42–83)	Pain‐shoulder	Cross sectional

SD = Standard deviation; TMD = Transcranial magnetic stimulation.

### Interventions/comparators

The search did not specifically target studies that had evaluated a specific intervention. However, six of the articles included reported data that had been collected as part of larger studies designed to evaluate interventions. Two studies presented data from control groups of intervention trials (Pandyan et al., 2003; Malhotra et al., 2011), one study presented data from both arms of a trial comparing day hospital and community‐based therapies (Wanklyn et al., 1996) and one study presented data from all cohorts in a study of antiplatelet therapy (O'Donnell et al., 2013). Two publications presented data from studies of the predictive value of motor evoked potentials (van Kuijk et al., 2007) and transcranial magnetic stimulation (De Jong et al., 2011).

### Outcomes

Table 2 summarizes outcomes measures and predictor variables used in the studies. Three of the studies briefly referred to the ease or difficulty with passive care of the arm (Lundstrom et al., 2008; Kong et al., 2010; Lundström et al., 2010). However, none of these studies measured this as an outcome in a systematic way, although increasingly, measures of difficulty with passive care of the arm are being developed (Bhakta et al., 2000). Fourteen of the publications examined spasticity after stroke, five considered contracture and 22 examined pain.

**Table 2:** Outcomes and predictor measures used in the studies

	Outcome measures	Predictors of impairment which were assessed		
Pain				
De Jong et al., 2011	MAS (elbow flexors)	Motor control (FMMA)		
Kong et al., 2012	AS (shoulder, elbow, wrist and fingers)	Stroke severity (NIHSS)	Global function (mod BI)	Weakness (UEMI)
		Sensation (MAND)		
Kong et al., 2010	AS (shoulder, elbow, wrist and fingers)	NA		
Kuptniratsaikul *et al.*, 2013	MAS (elbow and knee)	NA		
Leathley et al., 2004	Tone assessment scale	Higher cortical dysfunction	Global function (BI)	Weakness (3‐point scale)
Watkins et al., 2002	MAS (wrist, elbow)	(aphasia, confusion or inattention)	Side of stroke	Gender
			Premorbid function (mRS)	Diabetes
Lundstrom et al., 2010	MAS (shoulder, elbow, wrist, fingers, hip, knee and ankle)	Stroke severity (NIHSS)	Weakness (ssNIHSS)	Sensation (ssNIHSS)
Global function (mRS)				
Lundstrom et al., 2008	MAS (all arm and leg joints)	NA		
Moura et al. 2009	MAS (unclear which joint assessed)	Weakness (MST)	Gender	Age
		Pain (any report)		
Pandyan et al., 2003	MAS (wrist)	Arm function (ARAT)		
Picelli et al., 2013	MAS (shoulder, elbow, wrist and fingers)	Motor control (items of European Stroke Scale)		
Sommerfeld et al., 2004	MAS (all arm and leg joints)	NA		
Urban et al., 2010	MAS (all arm and leg joints)	Sensation (LT‐MAND)	Weakness (BMRC)	
Van Kujik et al., 2007	AS (elbow and wrist)	Arm control (FMMA)	Global function (BI)	Sensation (LT & FTT)
		Apraxia (clinical observation)	Inattention (MAND)	
Pain				
Appelros, 2006	Pain‐open question at assessment	Stroke severity (NIHSS)	Sensation (ss NIHSS)	Motor function (ss NIHSS)
Lundstrom et al., 2009	Pain‐VAS	None		
Sommerfeld and Welmer, 2012	Pain‐interview	Sensation light touch (perceiving touch with cotton wool)	Motor control (BL)	Global function (BI)
			Spasticity (MAS)	Proprioception (FTT)
Aras et al., 2004	Pain‐MAND	NA		
Bohannon 1988	Pain‐reported during examination	NA		
Cheng et al., 1995	Pain‐MAND	NA		
Gamble et al. 2002	Pain‐VAS	Mood (HADS)	Sensation (LT)	Global function (BI)
Gamble et al., 2000				
		Weakness (ssNIHSS)		
Hadianfard and Hadianfard, 2008	Pain‐VAS	Global function (Kenny)	Aphasia (any problem with	Visual field (MAND)
		Motivation (MAND)	speech)	Mood (symptom checklist)
		Sensation (NSAS and LT)		
Kuptniratsaikul *et al.*, 2013	Pain‐MAND	NA		
Lindgren et al., 2012	Pain‐VAS	Side of hemiplegia	Stroke severity (NIHSS)	
Lindgren et al., 2007	Pain‐VAS	Side of hemiplegia	Stroke severity (NIHSS)	
O'Donnell et al., 2013	Pain‐self report;	Stroke severity (NIHSS)	Gender	Depression (‘feeling sad’)
	Neurologist assessed the cause (MAND)	Alcohol intake (no. of drinks)	Smoker	Previous exercise
		Global function (mRS)		
Paci et al., 2007	Pain‐dichotomous response to pain at rest/ on mvt	Shoulder subluxation (palpation)	Motor control (FMMA)	Pain
Pong et al., 2012	Pain‐VAS	Motor control (BMR)	ROM (goniometer)	Sensation (MAND)
		Spasticity (AS)		
Poulin de Courval et al., 1990	Pain‐ reported during physical examination	NA		
Rajaratnam et al., 2007	Pain‐ numerical rating scale	NA		
Ratnasabapathy et al., 2003	Pain‐ questionnaire designed by study team	NA		
Roosink et al., 2011	Pain‐numerical rating scale at rest & on movement	NA		
Sackley et al., 2008	Pain‐ reported during physical examination	NA		
Suethanapornkul and Kuptniratsaikul, 2008	Pain‐ MAND	Global function (BI)	Subluxation (MAND)	Mood (HADS)
		Spasticity (MAS)	Motor control (Brunnstrom)	Cognition (Thai mental state exam)
		Proprioception (MAND)		
Wanklyn et al., 1996	Pain‐ questionnaire designed by study team	NA		
Zorowitz et al., 1996	Pain‐ VAS	NA		
Contracture				
Sackley et al. 2008	30% reduction in ROM (MAND)	NA		
Ada et al. 2006	ROM at elbow (measured from photograph‐ MAND)	NA		
Kwah et al., 2012	Torque‐controlled ROM at elbow wrist and ankle	Spasticity (Tardieu)	Stroke severity (NIHSS)	Motor control (Mot Ass Scale)
	All other joints‐ 4 point scale of movement restriction		Pain (NRS)	Strength (Manual muscle test)
Malhotra et al., 2011	ROM at wrist with standardized force	Arm function (ARAT)		
Pandyan et al., 2003	ROM wrist (goniometry with standard force)	Weakness (grip dynamometer)		

MAND = method of assessment not described; ARAT = action research arm test; AS = Ashworth scale; BMRC = British medical research council; BI = Barthel Index; BL = Birgitte Lindmark Motor Assessment; BMR = Brunnstrom motor recovery; FMMA = Fugl–Meyer motor assessment; FTT = Find the thumb; HADS = Hospital anxiety and depression scale; LT = light touch; MAS = Modified Ashworth Scale; Mod BI = Modified Barthel Index; MMSE = mini mental state exam; Mot Ass Scale = Motor assessment scale; mRS = Modified Rankin Score; MST = muscle strength test; NSAS = Nottingham Sensory Assessment Scale; NIHSS = National Institutes for Health Stroke Scale; ROM = range of movement; ssNIHSS = sub‐scale of NIHSS; UEMI = Upper extremity motor index; VAS = visual analogue scale.

Spasticity was most frequently measured with the Ashworth Scale, the Modified Ashworth Scale or Tone Assessment Scale, all of which grade the resistance to passive movement. Contracture was measured with a variety of methods including goniometry and photography, but not all studies described the methods used. Of the studies that examined pain, nine used a visual analogue or numerical scales, and one used a dichotomous variable (pain was either present or absent at rest or on movement). The remaining studies of pain either used unvalidated tools or did not stipulate the methods of its measure.

### Predictor measures

The studies examined a wide range of predictor variables including motor and sensory impairment, inattention, cognition, mood, global function and stroke severity (Table 2). Some used predictor measures that have well‐established validity and reliability such as the Barthel Index, while other studies developed their own means of assessing predictors often without reference to psychometric testing.

### Study designs

Characteristics of the study designs are summarized in Table 1. Twenty‐eight of the studies were longitudinal and six were cross‐sectional. All of the studies, with the exception of Pandyan et al. (2003) and Sackley et al. (2008), identified a single primary measure of a specific impairment after stroke and reported its incidence. Although a number of studies referred to evaluation of *predictors* of impairment, this term was interpreted in two different ways. Some studies followed a process where clinical tests were conducted at an early time point to then look at impact of these early predictors on disability or impairment in the longer term (for example, whether Barthel score at 7 days post‐stroke predicted longer‐term degree of spasticity). The remaining studies looked at the correlation between the selected outcome and related impairment at a single time point (for example, whether range of movement at a joint was correlated with pain). For this review, we included results that related only to early predictors and excluded reference to correlated impairments. A range of statistical analysis was used in the studies including univariate analyses, logistic regression and dividing participants into groups with specific impairments for comparison. In the synthesis of results, account was taken only of data related to incidence, change over time and evaluation of *early* predictors as these relate to the original research question.

### Quality assessment and risk of bias within studies

Inter‐rater agreement across reviewers for judging the quality of the studies was good with a kappa coefficient of 0.65 (Altman 1991). The areas of potential risk of bias identified in each of the studies are presented in Table 3. Methodological details reported in the papers were of variable quality. Most of the studies described selection criteria, but many restricted recruitment. The most common shortcomings related to inadequate assessor blinding (detection bias) (if comparing outcomes to predictors measures), and the use of unreliable or unvalidated data collection tools (performance bias). For example, three of the studies that considered pain did not state a consistent approach to its measurement (Cheng et al., 1995; Aras et al., 2004; Suethanapornkul and Kuptniratsaikul, 2008). A further nine used either visual analogue scales or numerical rating scales, and although these may be considered the best tools available, Price et al. (1999) demonstrated that people with stroke are often unable to accurately complete them. Given this, and the lack of formal protocol for assessing pain in the majority of studies, the measurement of this outcome is a potential area of bias in all of the studies that examined pain. Equally, there is some debate about whether the measures used to record spasticity, such as the Ashworth scale differentiate between the neural and muscular components of resistance, and studies of reliability and validity have shown mixed results (Fleuren et al., 2010). Nonetheless, they are widely used in both clinical practice and research trials.

**Table 3:** Potential risk of bias in included studies (positive response indicates less risk of bias)

	Is sample representative of target population?	Are assessors blinded?	Are data collection tools reliable and valid?	Are withdrawals reported?	Were participants unlikely to receive an unintended intervention?	Was statistical analysis appropriate?
Appelros, 2006	Yes	No	No	Yes	Yes	Yes
Ada et al., 2006	Yes	No	No	No	Yes	Yes
Aras et al., 2004	No	No	No	Yes	No	Yes
Bohannon 1988	Yes	No	No	Yes	Yes	Yes
Cheng et al., 1995	No	No	No	Yes	Yes	Yes
De Jong et al., 2011	No	No	Yes	Yes	Yes	Yes
Gamble et al. 2002	Yes	No	No	Yes	Yes	Yes
Gamble et al., 2000	Yes	No	No	Yes	Yes	Yes
Hadianfard and Hadianfard, 2008	Yes	No	No	Yes	Yes	No
Kong et al., 2012	No	No	Yes	Yes	No	Yes
Kong et al., 2010	Yes	No	No	Yes	No	Yes
van Kujik et al., 2007	No	No	Yes	Yes	No	Yes
Kuptniratsaikul *et al.*, 2013	No	No	No	No	Yes	Yes
Kwah et al., 2012	Yes	No	Yes	Yes	Yes	Yes
Leathley et al., 2004	Yes	No	Yes	Yes	Yes	Yes
Lindgren et al., 2012	Yes	No	No	Yes	Yes	Yes
Lindgren et al., 2007	Yes	No	No	Yes	Yes	Yes
Lundstrom et al., 2010	Yes	No	Yes	Yes	Yes	Yes
Lundstrom et al., 2009	Yes	No	No	Yes	Yes	Yes
Lundstrom et al., 2008	Yes	No	Yes	Yes	No	Yes
Malhotra et al., 2011	Yes	No	Yes	Yes	Yes	Yes
Moura et al. 2009	No	No	No	Yes	No	Yes
O'Donnell et al., 2013	No	No	No	Yes	Yes	Yes
Paci et al., 2007	Yes	No	Yes	Yes	No	Yes
Pandyan et al., 2003	No	No	Yes	Yes	Yes	Yes
Picelli et al., 2013	No	No	Yes	No	Yes	Yes
Pong et al., 2012	No	No	No	Yes	Yes	Yes
Poulin de Courval et al., 1990	Yes	Yes	No	Yes	No	Yes
Rajaratnam et al., 2007	No	No	No	Yes	Yes	Yes
Ratnasabapathy et al., 2003	Yes	No	No	Yes	Yes	Yes
Roosink et al., 2011	Yes	No	No	Yes	Yes	No
Sackley et al. 2008	Yes	No	No	No	Yes	Yes
Sommerfeld and Welmer, 2012	Yes	No	Yes	No	Yes	Yes
Sommerfeld et al., 2004	Yes	No	Yes	Yes	No	Yes
Suethanapornkul et al., 2008	Yes	No	No	No	No	Yes
Urban et al., 2010	Yes	No	Yes	Yes	Yes	Yes
Wanklyn et al., 1996	Yes	No	No	Yes	Yes	Yes
Watkins et al., 2002	Yes	No	Yes	Yes	Yes	Yes
Zorowitz et al. 1996	No	No	No	Yes	No	Yes

### Results of individual studies

Summary results of individual studies are presented in Tables 4, 5 and 6. For ease of interpretation, results are presented for distinct impairments and have been sub‐grouped into studies that recruited populations of all people with stroke against those who recruited only people with stroke who also had hemiplegia or weakness.

**Table 4:** Studies of spasticity: individual results

Study	Incidence of impairment	Reporting of change over time	Value of predictors
Studies which recruited a general population of people post stroke			
Kuptniratsaikul *et al.*, 2013	18% at 12 months	Not examined	Not examined
Leathley et al., 2004	36% at 12 months	Not examined	1. Any degree of spasticity predicted by
			↓ global function (*p* < 0.001)
			weakness (*p* < 0.001)
			2. Severe spasticity predicted by:
			↓ global function (*p* < 0.001)
			Right sided stroke (*p* < 0.02)
Watkins et al., 2002	Severe spasticity in 20% at 12 months		3. No relationship with higher cortical dysfunction, gender, diabetes and pre‐morbid function
Lundstrom et al., 2010	4% at up to 10 days, 27% at 1 month; 23% at 6 months	Not examined	1. Spasticity predicted by
			weakness (OR = 10: 95% CI: 2.1–48.4)
			stroke severity (*p* = 0.002)
			2. No relationship with sensation or global disability
Lundstrom et al., 2008	17% at 1 year	Not examined	Not examined
	6% had ‘disabling’ spasticity in the arm		
Moura et al., 2009	26% at final timepoint	Not examined	1. Spasticity predicted by
			pain (*p* < 0.0001; OR = 107.0; 95% CI: 13.5–847.3),
			weakness (*p* < 0.0001; OR = 91.9; 95% CI: 12.0–699.4)
			2. No relationship with gender or age
Sommerfeld et al., 2004	20% at 1 week, 18% at 3 months	Prevalence decreased over time	Not examined
Studies which recruited a population of people post stroke with hemiplegia or weakness			
De Jong et al., 2011	10% at 48 hours, 20% at 10 days, 42% at 3 months and 42% at 6 months	Some cases resolved at each time point with 1 new case at 6 months	Spasticity predicted by
			↓ motor control (*p* < 0.001)
Kong et al., 2012	33% at 3 months, 43% at 6 months and 47% at 1 year	Some cases resolved at 12 months, with some new cases at 6 and 12 months	1. Moderate to severe spasticity predicted by:
			↓ global function (*p* < 0.001)
	Severe spasticity in 17%		↓ motor control (*p* < 0.001)
			stroke severity (*p* < 0.001)
			2. No relationship with sensation
Kong et al., 2010	78%, severe in 38%	Not examined	Not examined
van Kujik et al., 2007	63% at any time point	Spasticity evident in 1 week, some cases resolved over all timepoints and few new cases at 26 weeks	No relationship between spasticity and arm control, global function, sensation, apraxia or Inattention
	55% at 26 weeks		
Pandyan et al., 2003	Not reported	Spasticity evident in 1 week, and developed over 32 weeks	Spasticity predicted by
			↓arm function (*p* < 0.01)
Picelli et al., 2013	44% had severe spasticity at 6 months	Not examined	Spasticity predicted by:
			↓ motor control (OR = 0.45 95% CI 0.31–0.65)
Urban et al., 2010	43%	Not examined	Spasticity predicted by
			weakness (*p* < 0.001)
	16% had severe spasticity		
			↓sensation (*p* < 0.001)

**Table 5:** Studies of pain: individual results

Study	Incidence of impairment	Reporting of change over time	Value of predictors
Studies which recruited a general population of people post stroke			
Appelros, 2006	11% reported any pain at 1 year	Not examined	Pain predicted by:
			stroke severity (OR = 1.24 95% CI: 1.11–1.39)
			weakness (OR 1.8 95% CI: 1.3–2.7)
			↓sensation (OR 3.2 95% CI: 1.5–6.5)
Gamble et al. 2002 Gamble et al., 2000	25% developed shoulder pain at 2 weeks; 40% developed shoulder pain within 6 months	80% of cases had resolved at 6 months	Shoulder pain predicted by
			↓sensation (*p* < 0.001)
			weakness (*p* < 0.001)
			No relationship with depression or global function
Hadianfard et al., 2008	32% reported shoulder pain within first year	6% reported shoulder pain in first 2 months, 12% within 4 months and 11% within 6 months	Shoulder pain predicted by
		Occasional case reported after 6 months	↓sensation (*p* < 0.0001)
			aphasia (*p* < 0.0001)
			↓ global function (*p* < 0.0001)
			depression (*p* < 0.001)
			↓motivation (*p* < 0.0001)
			No relationship with visual field deficit
Kuptniratsaikul *et al.*, 2013	34% reported shoulder pain at 12 months	Not examined	Not examined
Lindgren et al., 2012	22% reported shoulder pain within 4 months; 72% of these still had pain at 16 months	Few new cases at 16 months but resolved cases at all timepoints	Shoulder pain predicted by
Lindgren et al., 2007			stroke severity (*p* = 0.008)
			left hemiplegia (*p* = 0.01)
Lundstrom et al., 2009	21% report stroke pain at 1 year	Not examined	Not examined
O'Donnell et al., 2013	10.6% report chronic pain	Not examined	Chronic pain predicted by:
			Stroke severity (OR = 1.07 95% CI: 1.05–1.09)
			Previous depression (OR = 1.67 95% CI: 1.47–1.89)
			Previous alcohol intake (OR = 1.37 95% CI: 1.11–1.7)
			Diabetes mellitus (OR = 1.18 95% CI: 1.05–1.33)
			Peripheral vascular disease (OR = 1.44 95% CI: 1.09–1.91)
			Female sex
			Statin use
Rajaratnam et al., 2007	22% reported shoulder pain within 1 week	Not examined	Not examined
Ratnasabapathy et al., 2003	17% at 1 week, 20% at 1 month, 23% reported shoulder pain at 6 months	Pain presented within 1 week, 72% of cases had resolved at 6 months	Not examined
Sommerfeld et al., 2012	17% initially, 21% at 3 months, 17% at 18 months		Pain predicted by
			↓sensation (*p* < 0.05)
			↓mobility (*p* < 0.05)
			No relationship with spasticity, motor control or global function
Suethanapornkul and Kuptniratsaikul, 2008	19% developed shoulder pain	Pain resolved in 77% of cases	Pain predicted by:
			Shoulder subluxation (OR 2.06 95% CI: 1.08–3.95)
			No relationship with motor control, spasticity, proprioception, cognition, global function or mood

**Table 6:** Studies of contracture: individual results

Study	Areas of bias quality score (lower score = increased risk of bias)	Incidence of impairment	Reporting change over time	Value of predictors
Studies which recruited a population of people post stroke with hemiplegia or severe stroke				
Ada et al., 2006	3/6	51% of those with hemiplegia developed contracture	Contracture evident by 2 weeks and plateaued by 9 weeks	Not examined
Kwah et al., 2012	5/6	52% develop contracture	Not examined	Contracture predicted by
				stroke severity (*p* < 0.01)
				weakness (*p* < 0.01)
				↓motor function (*p* < 0.01)
				No relationship with pain or spasticity
Malhotra et al., 2011	5/6	100% of those without function develop contracture	Contracture evident by 6 weeks and plateaued by 24 weeks	Contracture predicted by:
				↓function (*p* < 0.01)
Pandyan et al., 2003	4/6	Not reported	Contracture evident by 6–8 weeks and developed over 32 weeks	Contracture predicted by
				Weakness (*p* < 0.01)
Sackley et al., 2008	3/6	43% had contracture at 3 months, 56% at 6 months and 67% at 12 months	Not examined	Not examined

## Synthesis of results

There were no studies that evaluated the natural course of development or potential predictors of difficulty caring for the arm after stroke in a systematic way. Three studies mentioned difficulty with hygiene and dressing (Lundstrom et al., 2008; Kong et al., 2010; Lundström et al., 2010). However, reference to these difficulties was included within qualitative interviews with an overall rating of difficulty with care, active function and mobility, so it was not possible to extract data related to passive care of the arm. Therefore, the synthesis only considered studies that had examined the related impairments of pain, spasticity and contracture. Because of the variation in reporting of data (most studies reported *p* values for predictors in isolation of other statistics), and heterogeneity of the included studies, a decision was made not to attempt meta‐analysis of the data. Therefore, the synthesis is narrative.

-
**Difficulty caring for the arm** There were no studies that evaluated the natural course of development or potential predictors of difficulty caring for the arm after stroke in a systematic way, although three studies mentioned difficulty with hygiene and dressing in a broader context. Kong, Chua *et al.* (2010) included interviews with people with stroke or their carers and identified ‘symptomatic spasticity’ as occurring when people reported difficulty with passive function, active function, pain or associated reactions. Lundström et al. (2010) and Lundstrom et al. (2008) defined ‘disabling spasticity’ as that which affected any movement, function or social experience and identified this from interviews and unstructured examinations. In all of these studies, difficulties with passive care were included within qualitative interviews, which also involved active function and mobility, so it was not possible to extract data related only to passive care of the arm. It is interesting to note that all of these studies aligned difficulty with passive care within the construct of ‘spasticity’, although a clear correlation between these constructs has not been established.
-
**Spasticity**

**Incidence** In studies that examined general populations of people post‐stroke, spasticity in muscles of the arm was present in 18% of participants at 3 months (Sommerfeld et al., 2004) and 17% at 1 year (Lundstrom et al., 2008). Populations of people who originally presented with weakness had a higher incidence of spasticity with rates between 33% at 3 months (Kong et al., 2012) and 78% at 12 months (Kong et al., 2010).

**Time course** Spasticity was evident in some participants as early as 48 hours post‐stroke (De Jong et al., 2011). Although the course of spasticity was fairly dynamic, for the majority of cases, it was evident in most participants who would experience it by 3 months (van Kuijk et al., 2007) and developed over at least 32 weeks (Pandyan et al., 2003). There were some cases where early spasticity resolved.

**Risk factors** The most frequent predictors of risk of spasticity were weakness (Lundström et al., 2010; Moura et al., 2009; Urban, Wolf *et al.* 2010; Leathley et al., 2004) and reduced motor control (Kong et al., 2012; De Jong et al., 2011; Pandyan et al., 2003). Stroke severity (Kong et al., 2012; Lundström et al., 2010) and reduced global function (Leathley et al., 2004; Kong et al., 2012) were also positive predictors of risk in at least two studies. The impact of sensory loss on spasticity risk is not clear, with one study identifying a positive relationship (Urban, Wolf *et al.*, 2010) and three discounting this (van Kuijk et al., 2007; Lundström et al., 2010; Kong et al., 2012). However, most of these studies did not clearly identify how sensation was quantified, making comparison difficult. Moura et al. (2009) identified early reports of pain as a predictor of risk of spasticity, but in this study, a significant number of areas of potential bias were identified on the quality assessment tool. Higher cerebral dysfunction including apraxia and inattention does not appear to increase risk (Leathley et al., 2004; van Kuijk et al., 2007).
-
**Pain**

**Incidence** Pain in any part of the body was reported by 10% (O'Donnell et al., 2013) to 21% of participants (Sommerfeld and Welmer, 2012) from a general population of people recovering from stroke, and incidence of shoulder pain occurred in 19% (Suethanapornkul and Kuptniratsaikul, 2008) to 40% (Gamble et al., 2002). Higher incidences of shoulder pain were found in studies of people with hemiplegia, or who were receiving rehabilitation. Within this population, incidence varied from 22% (Roosink et al., 2011) to 90% (Bohannon, 1988).

**Time course** Pain was reported as early as 1 week post‐stroke (Ratnasabapathy et al., 2003), with new cases of pain still being reported at up to 16 months post‐stroke (Lindgren et al., 2007). The highest incidence appeared to be within the first 6 months post‐stroke (Wanklyn et al., 1996; Hadianfard and Hadianfard, 2008). The course of pain was fairly dynamic, with some participants reporting resolution of pain at all time points over the first year post‐stroke (Wanklyn et al., 1996; Lindgren et al., 2007). However, one study found that 72% of people who experience shoulder pain at 4 months still had pain at 16 months (Lindgren et al., 2012).

**Risk factors** The most common predictor of increased risk of pain was reduced sensation (Gamble et al., 2000; Appelros, 2006; Hadianfard and Hadianfard 2008; Sommerfeld and Welmer, 2012), with shoulder subluxation (Paci et al., 2007; Suethanapornkul and Kuptniratsaikul, 2008), weakness (Gamble et al., 2000; Appelros, 2006) and stroke severity (Appelros 2006; Lindgren et al., 2007; O'Donnell et al., 2013) also identified as potential risk factors. The significance of depression was not clear, with two studies identifying a positive link with pain (Hadianfard and Hadianfard, 2008; O'Donnell et al., 2013), and two discounting this (Gamble et al., 2002; Kong et al., 2012). Equally, reduced global function was a predictor of pain in one study (Hadianfard and Hadianfard, 2008), but did not predict pain in two others (Gamble et al., 2002; Sommerfeld and Welmer, 2012). Aphasia and reduced motivation (Hadianfard and Hadianfard, 2008), and reduced mobility (Sommerfeld et al., 2004), had some predictive value in one study each. However, reduced motor control (Suethanapornkul and Kuptniratsaikul, 2008; Sommerfeld and Welmer, 2012), spasticity, proprioception and cognition (Suethanapornkul and Kuptniratsaikul, 2008) and visual field loss (Hadianfard and Hadianfard, 2008) were not associated with increased risk of pain.
-
**Contracture**

**Incidence** In a single study of a general population of stroke survivors, 52% of participants developed at least one contracture, with the most common joint affected being the shoulder (25%) and elbow (22%) (Kwah et al., 2012). In those with hemiplegia or severe stroke, 51% of participants had elbow contracture (Ada et al., 2006).

**Time course** Contracture was detected within 2 weeks of stroke (Ada et al., 2006) and continued up to 32 weeks (Pandyan et al., 2003), although only one study examined this time point.

**Risk factors** Contracture was most frequently predicted by weakness (Pandyan et al., 2003; Kwah et al., 2012) and reduced motor function (Malhotra et al., 2011; Kwah et al., 2012). It was linked with increased stroke severity but not degree of spasticity or pain (Kwah et al., 2012).

## Discussion

The purpose of this review was to identify the incidence and natural course of pain, spasticity, contracture and difficulty with passive care in the profoundly affected arm and to identify potential predictors of difficulty caring for the arm or these related impairments.

To date, there appear to be no studies that specifically examine the construct of difficulty caring for the profoundly affected arm after stroke. Although three of the studies identified in this review referred to difficulty with care of the arm (Lundstrom et al., 2008; Kong et al., 2010; Lundström et al., 2010), this was included within the construct of problematic spasticity and was identified in interviews and examinations along with difficulties with active function, pain and mobility. It was therefore not possible to identify the incidence or time course of difficulties caring for the arm as a discrete construct, or identify potential predictors of this problem. There is increasing recognition that in clinical practice, goals concerning the delivery of care to the arm are relevant, so future research in rehabilitation will need to examine this concept in detail.

Therefore, all of the studies included in this review focused on the impairments of spasticity, pain and contracture, which have been identified as having an association with difficulty caring for the arm. Risk of bias was fairly significant in most of the studies identified, particularly concerning the assessment tools used for quantifying both predictors and outcomes. Some studies included self‐developed tools with no reference to psychometric testing, but even those using recognized tools had some risk of bias, as many accepted tools still have limited validity and reliability (Hobart et al., 2007). Therefore, caution should be applied in drawing conclusions from the analysis.

There were higher incidences of pain, spasticity and contracture in people who originally presented with hemiplegia after stroke when compared with general populations of people recovering from stroke. In those with hemiplegia, the incidence of arm spasticity ranged from 33% to 78%, shoulder pain affected 22% to 90% and arm contracture was present in at least 50%. The incidence of both contracture and pain in the arm after stroke appears to be similar to that experienced by people following brain injury, where incidences of contracture between 44% (Yarkony and Sahgal 1987) and 84% (Moseley et al., 2008) and incidences of pain between 52% and 58% (Lahz and Bryant 1996) have been identified.

Spasticity and pain were detected from as early as 1 week after stroke, with contracture apparent by 2 weeks. Many cases were dynamic in presentation, with spasticity and contracture continuing to develop beyond 3 months post‐stroke and pain developing within 6 months of stroke. Therefore, clinicians may need not only to intervene early post‐stroke but also to be prepared to act over a longer time period in managing disability. As interventions are developed, they will also need to take account of the longer‐term evolution of these impairments.

Evaluation of the potential predictors of increased risk of impairment is limited, as many of the tools for quantifying the predictors were of limited quality. However, the most consistent risk factors for developing spasticity and contracture were weakness and reduced motor control, and the risk of pain is most commonly predicted by reduced sensation, shoulder subluxation, weakness and stroke severity. It is less clear if there is a relationship with higher cerebral functions and depression.

### Limitations of the review

A comprehensive literature search was undertaken as part of this review, but may be subject to retrieval bias. Notable omissions include the grey literature such as reports, conference proceedings and theses outside commercial publications and articles not published in English. In those studies that have been included, there are large variations within the populations of people with stroke studied, making synthesis of the results limited. Many of the studies themselves used data collection tools that may either not have been subjected to psychometric testing or, if they had, may still not be reliable in people with stroke with particular difficulties such as aphasia or inattention, adding further potential bias.

### Implications for physiotherapy practice

There is currently no evidence to predict the risk of developing difficulty caring for the profoundly affected arm after stroke. However, related impairments such as spasticity, contracture and pain affect a significant number of survivors and can start developing within 1–2 weeks of stroke and may not stabilize for at least 6 months post‐stroke.

There is no sufficient evidence for clinicians to develop targeted interventions at this stage. However, the research available suggests that clinicians may need not only to intervene early post‐stroke but also to be prepared to act over a longer time period in managing disability. Further research is required to establish the relationship between impairments and difficulty caring for the arm and to investigate if predictors of impairment can be used to identify those at risk of developing difficulty caring for the arm. This review has informed the design of a longitudinal study Care of the Arm after Stroke to test a range of predictors of difficulty caring for the arm and to develop a profile of impairment in people with profoundly affected arm.

## Ethical approval

No ethical approval was required.

## Conflict of interest

The authors report no declarations of interest.

## Supporting information

Supporting info item
