# Supplementary material for: Incidence, Time Course and Predictors of Impairments Relating to Caring for the Profoundly Affected arm After Stroke: A Systematic Review
Source: Physiother Res Int. 2015 May 25;21(4):210–27. doi: 10.1002/pri.1634 (PMC5157782; doi:10.1002/pri.1634)
Supplement: Supplementary file 1 — Supporting info item [file PRI-21-210-s001.docx]

**Appendix 1: Search terms: (Medical Subject Headings (MeSH) and key terms)**

| **Stroke related**  1. stroke  2. hemi*  3. 1 or 2  **Predictive**  4. predict*  5. risk  6. prognos*  7. longitudinal  8. course  9. 4 or 5 or 6 or 7 or 8  **Arm-related**  10. hand  11. arm  12. shoulder  13. wrist  14. upper extremity  15. 10 or 11 or 12 or 13 or 14 or 15  **Specific impairments**  16. passive function  17. spastic*  18. hyperton*  19. contracture  20. range of motion  21. pain  22. 14 or 15 or 16 or 17 or 18 or 19  23. 3 and 9 and 15 and 22 |
| --- |
